# Cysteine Modifications in the Pathogenesis of ALS

**DOI:** 10.3389/fnmol.2017.00005

**Published:** 2017-01-23

**Authors:** Cristiana Valle, Maria Teresa Carrì

**Affiliations:** ^1^Institute for Cell Biology and Neurobiology, CNRRome, Italy; ^2^Fondazione Santa Lucia IRCCSRome, Italy; ^3^Department of Biology, University of Rome Tor VergataRome, Italy

**Keywords:** amyotrophic lateral sclerosis, cysteine, neurodegeneration, protein aggregation, superoxide dismutase 1, TDP43

## Abstract

Several proteins are found misfolded and aggregated in sporadic and genetic forms of amyotrophic lateral sclerosis (ALS). These include superoxide dismutase (SOD1), transactive response DNA-binding protein (TDP-43), fused in sarcoma/translocated in liposarcoma protein (FUS/TLS), p62, vasolin-containing protein (VCP), Ubiquilin-2 and dipeptide repeats produced by unconventional RAN-translation of the GGGGCC expansion in C9ORF72. Up to date, functional studies have not yet revealed a common mechanism for the formation of such diverse protein inclusions. Consolidated studies have demonstrated a fundamental role of cysteine residues in the aggregation process of SOD1 and TDP43, but disturbance of protein thiols homeostatic factors such as protein disulfide isomerases (PDI), glutathione, cysteine oxidation or palmitoylation might contribute to a general aberration of cysteine residues proteostasis in ALS. In this article we review the evidence that cysteine modifications may have a central role in many, if not all, forms of this disease.

## Introduction

Amyotrophic lateral sclerosis (ALS) is an adult-onset fatal neurodegenerative disease characterized by fast progressing degeneration of upper and lower motor neurons of the motor cortex, brainstem and spinal cord. Motor neuron degeneration is associated with muscle weakness and atrophy followed by paralysis until death by respiratory failure (Robberecht and Philips, 2013). Although ALS is sporadic in the majority of cases (sporadic ALS, sALS), this disease is inherited genetically in a significant part of patients (familial ALS, fALS). ALS-associated genes code for proteins involved in diverse cellular processes, and diverse mechanisms have been proposed as major contributors to neurodegeneration in fALS and sALS (Renton et al., 2014). These include defective RNA metabolism, glutamate excitotoxicity, disruption of membrane trafficking, endoplasmic reticulum (ER) stress, mitochondrial dysfunction and protein misfolding and aggregation (Peters et al., 2015). The variety of these factors makes the etiology of the disease extremely complex, a fact that is reflected in the current unavailability of effective therapy.

A common feature observed in patients, regardless their classification as sporadic or familial, is the presence of motor neuronal inclusions, formed by misfolded aggregated proteins, which are associated with synaptic loss and neuronal death (Sasaki and Maruyama, 1994; Robberecht and Philips, 2013). In particular, patients carrying mutations in the genes coding for the antioxidant enzyme superoxide dismutase 1 (SOD1), for the RNA-binding proteins transactive response DNA-binding protein (TDP43) and fused in sarcoma/translocated in liposarcoma protein (FUS/TLS) or an expanded hexanucleotide GGGGCC in the *C9orf72* gene have proteinaceous inclusions made of, respectively, SOD1, TDP43, FUS and dipeptide repeats originating from RAN translation of the exanucleotide. Interestingly, TDP43 is found aggregated also in sALS and in non-TDP43 fALS patients, with the exception of those with SOD1 mutations (Lee et al., 2011).

In this article we review current evidence supporting the idea that ALS can be seen as a cysteninopathia following an incorrect redox state of cysteine residues.

## Cysteines in Oxidative Folding and in Cellular Redox Balance

Protein cysteine residues contain a thiol group that can form covalent disulfide bridges during the process of oxidative folding and thus are critical for correct protein structure, function and stability (Feige and Hendershot, 2011). In eukaryotic cells, stable intra-molecular or inter-molecular disulfide bridges are often formed in exported proteins in the oxidizing environment of the ER lumen (Walter and Ron, 2011; Oka and Bulleid, 2013) through reactions catalyzed by the family of protein disulfide isomerases (PDI; see below) or in the mitochondrial intermembrane space (IMS) for those proteins imported in this organelle through the MIA pathway (Mordas and Tokatlidis, 2015; Chatzi et al., 2016). Disulfide bridges exist also in cytosolic proteins, and chaperones such as the heat shock proteins Hps70 and Hps90 seem to be able to catalyze the formation of disulfide bonds and possess foldase activity in the more reducing cytosolic environment as well (Chambers and Marciniak, 2014).

Besides their role in disulfide bridging, cysteine residues also play a main role in maintaining a correct cellular redox balance. First, the cysteine residue of the tripeptide glutathione (GSH, γ-L-Glutamyl-L-cysteinylglycine) participates in a complex network of enzyme-catalized reactions (Meister, 1988). Glutathione is the major thiol antioxidant in mammalian cells and reduces disulfide bonds formed within cytoplasmic proteins by serving as an electron donor. In the process, glutathione is converted to its oxidized form, glutathione disulfide (GSSG), which can be reduced back by glutathione reductase, using NADPH as an electron donor. GSH serves as a cofactor for a number of antioxidant enzymes (such as glutathione reductases, glutathione peroxidases, glutathione S-transferases) that collectively collaborate to maintain a correct intracellular redox state and thus the ratio of reduced glutathione to GSSG within cells is often used as a measure of cellular oxidative stress (Meister, 1988).

Second, it is well known that redox-sensitive cysteine thiols are critical for signal transduction, transcription factor binding to DNA (e.g., Nrf-2, NF-κB), receptors activation and other processes (Jones, 2008). A clear overlap exists between signal transduction and redox biology, since the activity of enzymes in different pathways and transcription factors that work as redox sensors is based on disulfide bond formation, a mechanism that is often used to trigger and to maintain redox homeostasis (Forman, 2016).

## Cysteine-Dependent Aggregation and Mislocalization of ALS Proteins

Oxidative stress, that has been widely described in tissues obtained from ALS patients and transgenic mouse models (Cozzolino et al., 2008; Barber and Shaw, 2010), arises in conditions of unbalanced increase of reactive oxygen species (ROS) and reactive nitrogen species (RNS), which in turn may change the conformation of proteins and lead to the formation of aggregates and protein inclusions (Li et al., 2013). In the last 10 years, oxidation dependent, cysteine-mediated protein aggregation has been extensively demonstrated for mutant and wild-type SOD1 and TDP43.

Human homodimeric wild type SOD1 has four cysteine residues; two of them (Cys57 and Cys146) form an intra-monomer disulfide bridge, while Cys6 and Cys111 are un-bridged, with Cys111 relatively exposed on the protein surface near the dimer interface. The mechanism of mutant SOD1 aggregation involves oligomerization that may be the consequence of covalent disulfide cross-linking mediated mainly by Cys111 (Cozzolino et al., 2008). The Cys6 residue, which is packed tightly within the interior of the β-barrel, may play a role as well (Niwa et al., 2007), although all four Cys residues are mutated, and thus not present, in some patients^1^, which would argue against a direct role of Cys-mediated aggregation in the pathogenesis of ALS. On this line, data obtained in models *in vitro* and *in vivo* indicate that soluble forms of mutant SOD1 initiate disease and larger aggregates are implicated only in rapidly progressing events in the final stages of disease, and thus it has been argued that disulfide bond formation is a secondary effect and not primarily causative for aggregate formation in ALS (Karch et al., 2009). However, that article did not consider that uncontrolled accumulation of (aggregated) mutant SOD1 inside the mitochondria of cells may be directly responsible for mitochondrial impairment observed in ALS models and patients (Wiedemann et al., 2002; Ferri et al., 2006). Interestingly, cysteine residues are also involved in SOD1 localization in the IMS (Cozzolino et al., 2009; Kawamata and Manfredi, 2010). SOD1 import in IMS also involves its copper chaperone that acts in a redox-dependent manner, promoting SOD1 maturation through formation of disulfide bridges and its retention in this cellular compartment (Banci et al., 2008; Kawamata and Manfredi, 2010). Thus control of the redox state of cysteine residues and SOD1 aggregation in association with mitochondria may play a relevant role in the pathology of the ALS.

In line with this, alteration of the GSH/GSSG ratio may be a crucial trigger of the aggregation of mutant SOD1 and oxidized wild-type SOD1 (Ferri et al., 2006). In the light of the proposed toxicity of SOD1 oligomers and aggregates in ALS, possible strategies to counteract aggregation such as the modulation of the GSH/GSSG ratio (Ferri et al., 2006), the overexpression of cytosolic glutaredoxin 1 (Cozzolino et al., 2008) or mitochondrial glutaredoxin 2 (Ferri et al., 2010) and treatment with cisplatin (Banci et al., 2012), that binds Cys111, i.e. the crucial residue in SOD1 aggregation, have been tested. Although to different extent, all of these treatments were able to prevent or revert SOD1 aggregation in neuronal cells, ameliorating mutant G93A-SOD1 protein solubility, preserving mitochondrial function and preventing apoptosis, thus suggesting that modulation of the redox state of cysteine residues in specific compartments could be a significant therapeutic strategy for ALS. Interestingly, that glutathione deficiency leads to mitochondrial damage in the brain had been already reported in a seminal article by Alton Meister more than 25 years ago (Mårtensson et al., 1990) and it is known that GSH decreases with age (Ferguson and Bridge, 2016).

Recent experimental evidence highlighted similarities between the mechanisms of aggregation of SOD1 and TDP43. TDP43 has six cysteine residues, four of which (Cys173, Cys175, Cys198 and Cys244) are located in the two RNA recognition motifs (RRM1 and RRM2), while two others (Cys39 and Cys50) are in the N-terminal domain. No mutations in these residues have been reported so far^2^. Upon oxidative challenge, full length TDP43 (independently from the presence of ALS-linked mutations) is delocalized from the nucleus to the cytosol and forms both oligomers and large aggregates (Cohen et al., 2012; Bozzo et al., 2016). Studies on the aggregation process have shown that oxidation of cysteines located in the two RRMs decreases protein solubility, leading to the formation of intra and inter-molecular disulfide linkage (Cohen et al., 2012; Chang et al., 2013) and that cysteine residues in RRM1 direct the conformation of TDP43 (Shodai et al., 2013).

Formation of large aggregates is driven by oxidative stress and by partial unfolding of the hydrophobic core of the protein, whereas formation of oligomers depends on oxidative stress and clearly relies on accessible cysteine residues (Cohen et al., 2012; Shodai et al., 2013; Bozzo et al., 2016). The role of disulfide bridging as a main determinant of oligomers formation is further supported by the fact that oligomers are readily dissolved by reducing agents and by increasing available GSH (Bozzo et al., 2016), while depletion of the GSH pool induces insolubilization and fragmentation of wild type TDP43 in a motor neuron cell model (Iguchi et al., 2012).

Intriguingly, the two isoforms 35 kDa and 25 kDa deriving from the proteolytic cleavage of full length TDP43, that are found in the insoluble fraction in patients (Neumann et al., 2006) and may represent the truly toxic TDP43 species in mice (Walker et al., 2015), are totally included in cysteine-dependent oligomers (Bozzo et al., 2016).

Overall, since aggregates formed by SOD1 and TDP43 are basically present in all patients (including sALS), these data confer to a correct redox state of cysteine residues a pivotal role in the pathogenesis of ALS.

## Protein Disulfide Isomerases in ALS

PDIs are members of the thioredoxin superfamily and catalyze the formation, breakage and rearrangement of disulfide bridges of proteins via oxidation, reduction and isomerization reactions (Ellgaard and Ruddock, 2005; Rutkevich et al., 2010). While the disulfide interchange enzymatic activity involved in protein folding is their most relevant function in cells (Liu et al., 2005; Parakh and Atkin, 2015), PDIs can also act as molecular chaperones preventing aggregation of proteins whether they contain disulfide bonds or not (Cai et al., 1994) and this is possibly why genes coding for PDIs are among the main targets induced by the Unfolded Protein Response transcriptional program (Matus et al., 2013). A growing body of evidence suggests a role of PDI in the pathogenesis of ALS.

PDIA1 and PDIA3 (also known as ERp57) are up-regulated in spinal cords of SOD1^G93A^ mice, from pre-symptomatic to end stages of disease, and in tissues (spinal cord and peripheral blood mononuclear cells) from sALS patient (Atkin et al., 2006, 2008; Nardo et al., 2011). Moreover, PDIs are recruited to misfolded protein inclusions in sALS patients (Atkin et al., 2008) and interact with TDP43 and FUS inclusions in the tissues of ALS patients (Honjo et al., 2011; Farg et al., 2012). PDIs also co-localize with cytoplasmic aggregates in SOD1^G93A^ mice and in neuronal cells in culture (Atkin et al., 2006) and with mutant vesicle-associated membrane protein (VAMP)-associated protein (VAP) B (VAPB) *in vitro* (Tsuda et al., 2008).

That increased expression of PDIs in ALS represents an attempt to protection from toxic aggregates is further suggested by studies *in vitro* demonstrating that overexpression of PDI reduces mutant SOD1 inclusions, whereas silencing PDI expression increases their number, and that treatment of neuronal cells with (+/−)trans-1,2-bis(mercaptoacetamido)cyclohexane, an agent that mimics PDI activity, reduces mutant SOD1 inclusions in a dose-dependent manner (Walker et al., 2010; Jeon et al., 2014).

PDIs are usually localized in the ER; however, redistribution in vesicles seems to be related to the course of the disease (Walker, 2010). PDIs redistribution is associated with a significantly increased enzymatic activity and a reduction of the inactive S-nitrosylated PDIs form (Bernardoni et al., 2013; see below). Cellular redistribution of PDIs occurs via a process involving reticulons, a family of proteins devoted to the maintenance of the ER curvature. Overexpression of reticulon-1C (Rtn1-C) or reticulon-4A (NogoA) induces a new localization of PDIs in an ALS neuronal cell model and knockdown of NogoA accelerates motor neuron degeneration in SOD1^G93A^ transgenic mice (Yang et al., 2009; Bernardoni et al., 2013). This suggests the importance of a non-ER location of PDIs as a possible protective factor in ALS.

However, PDIs accumulation at the ER-mitochondria junction triggers apoptosis via mitochondrial outer membrane permeabilization pore (Hoffstrom et al., 2010; Zhao et al., 2015) and detrimental activities of PDIs in this location were identified in rat models of Huntington’s disease and Alzheimer’s disease (Sun et al., 2006; Hoffstrom et al., 2010). Similar observations in ALS models have not yet been reported; however, this aspect of PDIs’ biology would certainly deserve attention since the mitochondria-associated ER membranes (MAMs) seem to be a critical cellular compartment in ALS. In two recent elegant articles, Miller and his group have reported that localization of both TDP43 and FUS in MAMs activates GSK-3β to disrupt the VAPB–PTPIP51 interaction and in turn ER–mitochondria associations (Stoica et al., 2014, 2016).

A further evidence of a crucial role of cysteine metabolism in ALS comes from the recent discovery of PDI mutations in patients. Intronic variants of the gene encoding PDIA1 were reported to be a genetic risk factor for sALS and fALS (Kwok et al., 2013; Yang and Guo, 2016) and nine PDIA1 missense variants and seven PDIA3 missense variants were documented in 16 ALS patients (Gonzalez-Perez et al., 2015). Expression of ALS-linked mutant forms of PDIA1 and PDIA3 in a zebrafish model impairs synaptic proteins expression and determines motor neuron morphology alterations (Gonzalez-Perez et al., 2015). Moreover, *in vitro* dendritic outgrowth is decreased when ALS-linked PDI mutants are expressed (Gonzalez-Perez et al., 2015). Interestingly, mice knockout for PDIA3 in the nervous system show neuro-muscular junction deficit, impaired motor performance and reduced expression of synaptic vesicle transporter protein (Woehlbier et al., 2016).

Altogether these results strongly suggest that PDIs mutations explicate their pathological effects through a loss of function mechanism, which is consistent with the report of inhibition of PDI enzymatic activity by aberrant S-nitrosylation in patients and murine experimental models (see next paragraph).

## Redox-Dependent Post-Translational Modifications of Cysteines

Cysteine-dependent modifications of proteins are the most abundant post-translational modification taking place in an oxidative and/or nitrosative stress context and are considered an important mechanism of control of signal transduction.

Among these modifications, *S-nitrosylation*, a covalent addition of a NO group to a cysteine thiol, is generally a reversible modification (Hess et al., 2005) that may become irreversible in pathological conditions such as neurodegeneration (Nakamura et al., 2013). S-nitrosylation of PDIs has been found in several neurodegenerative disorders including ALS (Chen et al., 2013; Nakamura et al., 2013). When PDIs undergo S-nitrosylation in the active site, their enzymatic activity is inhibited and their protective functions are reduced (Benhar et al., 2006). In post-mortem spinal cord from sALS and fALS patients S-nitrosylated PDIs levels are five-fold more abundant compared to healthy controls and similarly high levels are detected in transgenic SOD1^G93A^ mice (Walker et al., 2010). Moreover, S-nitrosylation of PDIs increases insoluble aggregates of mutant G93A-SOD1 in spinal cord of transgenic mice (Chen et al., 2013; Jeon et al., 2014). Decreased S-nitrosylation has been associated to ALS as well. A subset of fALS patients with SOD1 mutations show an increased denitrosylase activity of *S*-nitrosoglutathione reductase (GSNOR) and this increase has been observed also in neuronal cells expressing the same mutant SOD1 (Schonhoff et al., 2006). Moreover, GSNOR up-regulation confers resistance to NO-releasing drugs in cells expressing mutant G93A-SOD1 (Rizza et al., 2015). Because of the known impact of S-nitrosylation on mitochondrial function (Di Giacomo et al., 2012), we can speculate that modulation of S-nitrosylation and the differential accessibility of cysteines may contribute to ALS pathogenesis.

*S-glutathionylation* is another reversible post-translational modification induced by ROS/NOS which results in the formation of a disulfide bond between GSH and a cysteine residue of proteins (Xiong et al., 2011). This modification is involved in the regulation, through a redox signal transduction mechanism, of different enzymes implicated in cellular homeostasis e.g., in signaling pathways, antioxidant response, energy metabolism and protein folding (Mieyal et al., 2008; Grek et al., 2013). A detrimental role for S-glutathionylation in ALS has been reported. For instance, S-glutathionylation on Cys111 induces dissociation of wild type- and fALS mutant G93A-SOD1 dimers (Redler et al., 2011), triggering monomer formation and subsequent aggregation (Wilcox et al., 2009; McAlary et al., 2013).

Finally, a growing body of evidence suggests that also *cysteine palmitoylation* could be implicated in ALS. Palmitoylation is the only lipid modification that can be reversibly regulated; its main role seems to be to constitute rafts that allow the dynamic targeting of specific proteins to membranes (Levental et al., 2010). It was observed that Cys6 can be palmitoylated in wild type SOD1 and that two fALS SOD1 mutants are more exposed to this change in motor neuronal cells and in the spinal cord of SOD1^G93A^ transgenic mice (Antinone et al., 2013). Moreover, palmitoylation takes place mainly on reduced disulfides, suggesting that immature SOD1 is the species primarily subject to this modification, and therefore increased when the cysteine residues are more exposed as observed for several mutant SOD1s (Antinone et al., 2013).

## Conclusions

**Figure 1:**
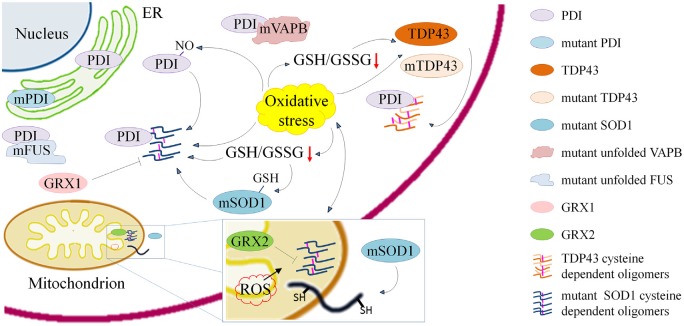
**Schematic representation of processes involving cysteine residues and that are relevant in the pathogenesis of amyotrophic lateral sclerosis (ALS) either through the induction of misfolding, aggregation and delocalization of proteins or through their inactivation.** Cysteine dependent protein aggregation in ALS is promoted by oxidative stress and reduced γ-L-Glutamyl-L-cysteinylglycine (GSH)/glutathione disulfide (GSSG) ratio. Wild type and mutant transactive response DNA-binding protein (TDP43) form cytoplasmic oligomers based on the accessibility of cysteine residues. Mutant superoxide dismutase 1 (SOD1) forms cytoplasmic and mitochondrial oligomers that can be reduced by overexpression of anti-oxidant proteins cytosolic Glutaredoxin 1 and mitochondrial Glutaredoxin 2. Cys-glutathionylation of mSOD1 and Cys-nitrosylation of protein disulfide isomerase (PDI) enhance aggregation of mSOD1. ALS associated PDI mutations suggest a crucial role of the cysteine-mediated folding in the disease. PDIs colocalize with mSOD1 and TDP43 oligomers and with mutant fused in sarcoma (FUS) and vesicle-associated membrane protein (VAMP)-associated protein (VAP) B (VAPB).

As outlined above, dysregulation of the redox state of cysteines seems to be involved in a number of mechanisms that are important for the maintenance of correct protein folding and activity in ALS (Figure 1 and Table 1). Other aspects of cysteine metabolism may be relevant in the pathogenesis of ALS, such as the direct oxidation of cysteines in proteins that are crucial for motor neuron metabolism and survival. One example is cysteines oxidation in AMP-activated protein kinase (AMPK) that is known to increase its activity (Zmijewski et al., 2010; Cardaci et al., 2012; Jeon and Hay, 2015). Intriguingly, an increased AMPK activity was reported in motor neuron cells expressing mutant SOD1 or TDP43 (Lim et al., 2012; Perera et al., 2014; Sui et al., 2014; Liu et al., 2015), in embryonic neural stem cells derived from SOD1^G93A^ mice (Perera et al., 2014) and in motor neurons of sALS and fALS patients (Liu et al., 2015). Moreover, pharmacological inhibition of AMPK activity rescued TDP43 mislocalization in neuronal cells and delayed disease progression in TDP43 transgenic mice (Liu et al., 2015) whereas genetic reduction of AMPK ortholog improved locomotor behavior and fecundity of *C. elegans* expressing G85R-SOD1 or M337V-TDP43 (Lim et al., 2012).

**Table 1:** **Involvement of cysteine residues in amyotrophic lateral sclerosis (ALS)**.

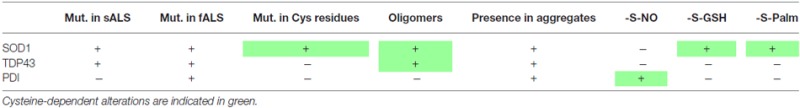

On the whole, studies exploring the possibility to modulate the redox state of cysteines are warranted with the aim of finding new therapeutic approaches for this disease. While genetic modulation of proteins involved in cysteine homeostasis is still an unfeasible approach in man, pharmacological interventions (e.g., to increase GSH/GSSG ratio or PDI activity) may hold great promise in the treatment of ALS.

## Author Contributions

CV and MTC formulated the entire concept of manuscript. CV executed complete drawing of figure. Both authors reviewed the manuscript.

## Conflict of Interest Statement

The authors declare that the research was conducted in the absence of any commercial or financial relationships that could be construed as a potential conflict of interest.

## Footnotes

^1^http://alsod.iop.kcl.ac.uk/

^2^http://alsod.iop.kcl.ac.uk/

## Abbreviations

ALS: Amyotrophic lateral sclerosis
AMPK: AMP-activated protein kinase
ER: endoplasmic reticulum
fALS: familial amyotrophic lateral sclerosis
FUS/TLS: fused in sarcoma/translocated in liposarcoma protein
GSH: reduced glutathione
GSNOR: S-nitrosoglutathione reductase
GSSG: oxidized glutathione
IMS: internal mitochondrial space
MAM: mitochondria-associated ER membranes
PDI: protein disulfide isomerase
RNS: reactive nitrogen species
ROS: reactive oxygen species
RRM: RNA recognition motif
sALS: sporadic amyotrophic lateral sclerosis
SOD1: Cu,Zn superoxide dismutase
TDP43: transactive response DNA-binding protein
VAPB: vesicle-associated membrane protein (VAMP)-associated protein (VAP) B.
